# Restricted access data in the neurosciences: Are the restrictions always justified?

**DOI:** 10.3389/fnins.2022.975795

**Published:** 2023-01-24

**Authors:** Richard Lathe

**Affiliations:** Division of Infection Medicine, University of Edinburgh Medical School, Edinburgh, United Kingdom

**Keywords:** falsifiability, open access, health data, replicability, sequence data, psychological data, imaging data, microbiome data

Personal healthcare information has historically been protected in law through physician–patient privilege, and recent legislation in the USA and the EU has sought to impose firm restrictions on sharing of such information. However, data access is a fundamental aspect of scientific research, and there is increasing conflict between data security/anonymity and the concepts of falsifiability and open science. This conflict is discussed from the perspective of the neurosciences (including cognitive science and experimental psychology) where researcher access to relevant data, even if anonymous, is increasingly constrained. In this age of ‘data protection', restricted access to personal data has popular appeal. But is it always justified? Epidemiological data are a special case because maximizing their utility constrains anonymization, and restrictions on access may be necessary. However, do we include anonymous genomic/transcriptomic sequence information, brain imaging data, brainwave recordings, eye-tracking data, body-posture recordings, Rorschach tests, or even microbiome studies? There is no evidence to date that, if properly anonymized, the identity of any individual can be deduced from such data (unless relevant data are already on the internet - ‘chicken and egg’). With the exception of epidemiological data, it is argued that objective evaluation is needed, and that restrictions on sharing anonymized datasets of other types should either be empirically based or set aside.

## Introduction

Historically, data pertaining to health and healthcare have always been considered to be confidential information that is shared only between physician and patient—*le secret medical*—paralleling the legal concept of attorney–client privilege (Shuman, 1985). The General Medical Council in the UK, for example, has decreed that patient information may only be disclosed with the explicit (or implied) consent of the patient, or in special circumstances such as notification of infectious diseases (Rimmer, 2017). However, two developments complicate the situation. First, electronic health data are increasingly filed online such that a consulting physician, perhaps even in another town, can access accurate patient records for the specific purposes of diagnosis or treatment. Second, the emergence of computerized data pertaining to sequence and other related data has raised the worrying prospect, among others, of “genetic discrimination,” for example by insurance companies—where insurers might demand access to genomic or other data to evaluate the presence of disease-causing biomarkers.

Regarding the former, many countries have considered that the existing legal framework regarding the confidentiality of health data is already adequate. In the latter case, some countries have adopted specific legislation to prevent or limit insurer use of genetic data, whereas others have relied on existing legal guidelines (Rothstein and Anderlik, 2001; Joly et al., 2003, 2014). However, in both the USA and Europe further legislation has been passed that restricts access to personal data in ways that challenge scientific research.

In the EU, Article 4 of the General Data Protection Regulation (GDPR) (European Union, 2019), stipulates that personal data are restricted—“personal data” means any information relating to an identified or identifiable natural person (“data subject”); an identifiable natural person is one who can be identified, directly or indirectly, in particular by reference to an identifier such as a name, an identification number, location data, an online identifier or to one or more factors specific to the physical, physiological, genetic, mental, economic, cultural or social identity of that natural person.

The situation in the USA is somewhat less clear. The Health Insurance Portability and Accountability Act of 1996 (HIPAA) permits disclosure of “deidentified” data and strives to strike a balance that permits important uses of information while protecting the privacy of people who seek care and healing (US Department for Health and Human Services, 1996; Centers for Disease Control and Prevention, 2003). HIPAA specifically restricts “individually identifiable health information” but contains some exemptions. These include judicial proceedings and research (“under certain conditions”).

The lack of clarity of what may and may not be disclosed has led to “erring on the side of caution.” Although we all agree that personal data must be protected, we are now facing situations where—irrespective of privacy issues—there are bans on sharing of otherwise anonymous data that now constrain many types of research. GDPR has “complicated the operation of research biobanks... without appreciably improving privacy protections” (Peloquin et al., 2020 for discussion); GDPR has blocked at least 40 international studies on cancer, and other collaborative projects are similarly threatened (Eiss, 2020). Although well-intentioned, the inferred need to withhold some types of data conflicts with two basic principles.

## Two principles of scientific endeavor: Falsifiability and open science

A basic principle of scientific investigation was put forward by Karl Popper in 1934 in his *Logic der Forschung* (Popper, 1935)—“falsifiability”—that demarcates a scientific statement from other types of assertions. Verifiability, reproducibility, refutability, testability, and empirical support are lumped together here under the generic term “falsifiability,” although this is an oversimplification: Gezelter argues that only falsifiability is inductively valid (Gezelter, 2022), whereas LeBel argues for replicability (LeBel et al., 2017), of note given the so-called replicability crisis in experimental psychology (e.g., Romero, 2019). However, irrespective of the term we apply, scientific statements must be based on empirical evidence and independent scrutiny. Therefore, if the primary data are not openly available (irrespective of the reason), then any conclusions based on those data become unfalsifiable, and thus fail the demarcation test.

This also applies to peer review. If the reviewers of a scientific paper are not able to access the primary data, then it is not possible for them to offer an opinion on whether the interpretation is correct.

Such considerations (among many others) have fueled calls for open science (Eckersley et al., 2003). Many funding bodies including government research organizations have determined that research data must be shared openly. The 2016 UK Concordat on Open Data, which, although accepting that there may sometimes be legitimate reasons to defer release of data, stipulates in its guiding principles that “Open access to research data is an enabler of high-quality research,” and that “Researchers will, wherever possible, make their research data open and usable within a short and well-defined period” (Research Councils UK, 2016; UK Concordat Subscribers, 2016), a view consonant with the 2016 Amsterdam Call for Action on Open Science (Netherlands EU Presidency 2016 Experts and Stakeholders, 2016).

For the US National Science Foundation (NSF), where it is also mandatory to collect publicly funded data for dissemination (Burwell et al., 2013), it is stated that “Open data should be made available to the widest range of users for the widest range of purposes” (National Science Foundation, 2016). The Swiss National Science Foundation (SNSF) states “Research data should be freely accessible to everyone—for scientists as well as for the general public” (Swiss National Science Foundation, 2022). A recent decision from the US National Institutes of Health (NIH) recognizes that “Sharing scientific data accelerates biomedical research discovery, in part, by enabling validation of research results, providing accessibility to high-value datasets, and promoting data reuse for future research studies” (National Institutes of Health, 2020). This edict will require scientists not only to share data on widely accessible websites at time of publication, but will also require researchers to develop a “Plan” for datasharing early in the process of grant submission. The guidelines will apply from January 2023.

Despite these clear imperatives, can we share anonymized information? To date there is no consensus.

## What are anonymized data?

There is no agreement on when data are considered to be “anonymized” (Eiss, 2020). There is an excellent plain language discussion by Finnegan and Hall who clarify various terms such as “anonymization” and “pseudo-anonymization” (Finnegan and Hall, 2017). The commentary by Peloquin et al. (2020) is also very valuable in this context (pp. 698–699). Further insightful debate on the underlying principles can be found in Chevrier et al. (2019) and Olatunji et al. (2022). The basic strategy for anonymization is to remove all personal details including specific identifiers such as name, date of birth, and address (“de-identification”), but this may be insufficient to preclude identification (see below). One approach is to dilute or blur the data to a point where re-identification is impossible (discussed in Goldacre and Morley, 2022) through strategies such as “obfuscation.” It is unlikely, for example, that an exact birth date is necessary, and current age is probably satisfactory for most research purposes. Nevertheless, there are instances where “anonymity” can be broken (below).

## *k*-mer data

There is a real risk that some data “on its own does not identify individuals, but could do so were it to be linked to other information” (Caldicott, 2013). Even with entirely anonymized data, one dataset (anonymous) can find matches in another. The term “*k*-mer” refers to a small number, *k*, of parameters that may be sufficient to identify specific individuals from anonymized data through a process of “re-identification” (Porter, 2008) (https://en.wikipedia.org/wiki/Data_re-identification). For example, Latanya Sweeney established that the majority of people in the USA can be uniquely identified by the combination of ZIP code, birth date, and sex (Sweeney, 2000). The *k*-mer issue has been extensively discussed and potential remedies debated (Sweeney, 2002; Ohm, 2010; Sweeney et al., 2018; Goldacre and Morley, 2022).

## Existing data: The chicken and egg (C&E) issue

The issue of pre-existing data availability has not been as extensively debated, and the term “C&E” is used here as a handy soubriquet. “Which came first, the chicken or the egg?” is a common query that relates to interdependence of two items. How this might apply to data confidentiality is amply illustrated by photographs of people. Using an anonymous photograph of an unknown individual, it is possible to identify him/her by searching on the internet (https://www.wikihow.com/Search-and-Find-About-Someone-Using-Image-Easily#Using-Google-Image-Search). This searches for other images that are identical or nearly identical to the same image, and (particularly for pictures of celebrities) multiple (correct) matches can be found. However, searches for an image not previously recorded find multitudes of supposed “matches” to images of unknown people (sometimes 50% of a different gender; unpublished observations); in these cases a photograph alone does not disclose their identity. In other words, the search is only successful if the identical or near-identical image is already available *via* the internet.

In general terms, the C&E issue can be summarized as follows: given one dataset (e.g., the egg) it is possible to identify the cognate chicken—but only if the chicken data are already available (or *vice versa*). This consideration applies to all types of data—if sufficiently detailed, all anonymous biomedical/physiological/psychological data can be uniquely matched to other relevant datasets, but only if these are in the public domain.

It could therefore be argued that data release is legitimate if the same data are already openly available. Nevertheless, GDPR “personal data” refers to any person who can be identified by (any) factors specific to (that) natural person. GDPR would therefore appear to cover an anonymous photograph that is already available in identifiable form on the internet, although this is unlikely to have been the intended aim of the legislation.

## Epidemiological data: Data anonymization is not always robust, and full anonymization may be counterproductive

Epidemiological data have been invaluable in understanding how different medications and lifestyles modulate, for example, the risk of neurocognitive disorders. Even with the largest dataset, all irrelevant details can in principle be deleted to avoid the *k*-mer problem, and personal identifiers can be replaced by an automatically generated code to prevent identification of individuals. If need be, encryption techniques can be deployed (e.g., Shiota et al., 2011). However, there are issues with removing all identifiers from epidemiological data. This is for two reasons.

First, although in some countries (e.g., Denmark and Taiwan) data are available in a single database, in most other countries different types of data are held in separate databases. A central challenge is to accurately match entries to each other. For example, in evaluating medication efficacy (or risk), patient outcomes in terms of disease diagnosis and severity (held in one database) need to be matched to medication/pharmacy records (often held in a second database). Complete removal of personal details constrains this—especially if different anonymization codes are applied to different datasets, where a single coding difference (e.g., one entry is removed from dataset A because it is empty, but is retained in dataset B) could prevent extraction of any meaningful information.

Second, there is a need to correct for confounds such as social deprivation and/or socioeconomic status (often based on address or ZIP code) that can have large effects on disease susceptibility. Again, removal of key information makes this difficult.

Third, there is a real risk that subjects who are made aware that their data may be used for research purposes may be less inclined to participate, and steps may need to be taken to provide reassurance of the scope and objectives of the release of any data.

## Restrictions on epidemiological data in neuroscience

For these and other reasons, many restrictions are in place regarding release of epidemiological data. Countries from Denmark to Taiwan have a total bar on sharing data from national epidemiological databases with researchers abroad. Individual countries have imposed restrictions on their own researchers. In Scotland, only researchers who have completed a validated course of instruction can be given access to healthcare data, and they must be listed on a “National Register of Approved Researchers” (Scottish Government, 2015). The curtain of red tape is formidable: “Researchers told us of their concern about the complexity, confusion and lack of consistency in the interpretation of the requirements they have to satisfy before research projects can proceed” (Caldicott, 2013). It can take months to years to gain authorization. In one case, research funding for data analysis in a neuroscience project was awarded, but the grant expired before access authorization was received—despite assurance of data access from the data repository (personal experience). Similar frustrations have been voiced elsewhere (Filippon, 2015).

Even once access is authorized, there is a legal barrier to studying the evidence because all such data must be held in “data havens” (Burton et al., 2022). These are high-security vaults that are generally only accessible to employees of the institution harboring the “data haven.” In technical terms, in Scotland these are “electronically Secure Analytic Platforms in physically secure data centers, with access provided either from a ‘Secure Safe Setting'... or *via* a Virtual Private Network or encrypted communication sessions” (Scottish Government, 2015).

Thus, irrespective of accreditation of various types, >99% of researchers cannot check any analysis based on these restricted data simply because they do not have access to (or even know how to access) a “data haven.” The conclusions of most epidemiological studies therefore cannot be independently validated by reference to the same original dataset. When a prominent analytics team was asked to validate work done by another team, it was found that “the analysts cannot replicate the work” (Goldacre and Morley, 2022) because the codelists (presumed to be the diagnostic codes) and other data were unavailable for scrutiny.

However, epidemiological data may be a special case where restrictions are justified. This is most unfortunate from many perspectives, but data havens might be a “necessary evil,” at least for epidemiological data, and we may need to live with them. The multiple issues are extensively debated in the recent “Goldacre Review” which recommends that proper investment in data curation will be essential to make epidemiological data rapidly accessible (Goldacre and Morley, 2022).

The downside is, of course, that few of the epidemiological data we read in current medical journals have been (or can be) independently validated by reference to the same dataset, and are therefore not falsifiable.

## Other types of data: Genomic data with restricted access

The First International Strategy Meeting on Human Genome Sequencing (Bermuda, 25–28 February 1996) agreed that “all human genomic sequence information, generated by centers funded for large-scale human sequencing, should be freely available and in the public domain in order to encourage research and development and to maximize its benefit to society” (HUGO, 2003).

However, the discovery by the relatives of Henrietta Lacks that the genomic sequence of the famous HeLa cell line (that was obtained without the consent of the donor; Editorial, 2020) led to ramifications because this was a disclosure of the data of a very specific individual, with potential medical implications for her relatives who would share some of the sequences. Henrietta's daughter is quoted as saying “I look at it as though these are my grandmother's medical records that are just out there for the world to see” (Callaway, 2013). Nevertheless, following consultation the family agreed to make the data available to researchers for biomedical research only (Callaway, 2013).

This precedent, and the fear of breaching data protection guidelines, has led to draconian restrictions on the release of even fully anonymized datasets that could not be used to identify specific individuals. For example, the US National Institutes of Health (NIH) and the National Institute for Biotechnology Information (NCBI) have issued stern restrictions on release of genomic information (https://ops.od.nih.gov/scientific-sharing/genomic-data-sharing/) because of the (inferred) risk of identifying individuals.

However, is this justified? Jablonka and Lamb observe “Many non-geneticists believe that knowledge of a person's complete DNA sequence will enable all their characteristics to be known and their problems predicted. This widespread belief in ‘genetic astrology’ leads to many unrealistic hopes and fears” (Jablonka and Lamb, 2007). Could any personal details be inferred from an anonymous genomic sequence alone? We can deduce the sex of an individual, and possibly his/or her racial origins, although that is not always easy, and this falls short of identifying an individual. Facial morphologies are often conserved between identical twins, and must therefore be largely encoded in the genome, but there is no means to generate an accurate “picture” of an individual from his/her genomic sequence. Even if it became possible in the far distant future to generate a fuzzy picture, this same profile would be shared by thousands if not millions of individuals (there are almost 8 billion of us in the world at time of writing), and this of course does not tell us anything of his/her personal details, such as date of birth, occupation or address.

As always in this field, there are complexities. First, it has been reported that it may be possible to identify anonymous participants (i.e., genomic sequence donors) by cross-reference to genealogy DNA databases (Callaway, 2013) (the C&E problem again).

Second, the Combined DNA Index System (CODIS, established in 1990) and the National DNA Index System (NDIS, in 1998) in the USA maintain records of DNA profiles, populated principally from crime scenes, consisting of short tandem repeats (STRs) at a set of about 20 core loci (Butler, 2006). These data are not publicly available. However, in the USA the authorities have the right to search ancestry databases (where millions of individuals have uploaded their genomic data) in criminal proceedings (Kaiser, 2019). The UK National DNA Database (established in 1995) also only comprises STR records that are held securely.

Forensic STRs are short repeated sequences that are highly variable among individuals, and STR profiles can often be sufficient to uniquely match (or at least satisfy a court of a match) an individual suspect to a sample retrieved from a crime scene, or to provide matches for the purposes of paternity/maternity testing. However, an STR profile alone is unable to identify an individual, and also cannot be used to predict a phenotype (e.g., disease risk) (Wyner et al., 2020). Forensic STR data are also predominantly from intergenic regions, and cannot be picked up by RNA-seq.

Nevertheless, using the STR approach it is in principle possible to identify a person within some genomic datasets using only very small amounts of genomic data. However, this requires access to the DNA data of the person to be identified (C&E problem). If one only has the sequence of the egg, it is not possible to identify the corresponding chicken (there are currently 30 billion chickens in the world, and nearly 8 billion humans).

Other potential methods to infer the identity of a person from a genomic sequence are discussed by Finnegan and Hall (2017), but these are extremely complex and require specialized tools and knowledge. Although subject to debate, some potential methods appear to be technically flawed and “do{es} not really identify anyone” (e.g., Ehrlich, 2017).

Overall, “Genomic data do not sit comfortably within the current legal and regulatory framework as a consequence of their nature and an overall lack of regulatory coherence” (Finnegan and Hall, 2017), and there is an ongoing need to study whether anonymized genomic information can really be used to identify a specific individual. If this proves to be immensely difficult (beyond the routine resources of even government agencies), then pragmatism argues that genomic data, appropriately anonymized, ought to be eligible for release to researchers.

## Transcriptomic data

Even though inferring the identity of an individual from (anonymized) DNA sequence data is fraught with difficulty, the restrictions are being expanded to include tissue RNA sequencing (RNA-seq) data, for example from brain. Thousands of human RNA-seq datafiles are already publicly available for scrutiny and are accessed by researchers worldwide on a daily basis, with no evidence of “re-identification,” adverse consequences, misuse, or breach of data protection. The Gene Expression Omnibus (GEO) team at NCBI states “there are no restrictions” on filing such data. Indeed, the majority of brain RNA-seq datasets are from post-mortem samples, further dampening the risk of misuse (discussed further below).

By contrast, RNA-seq data held at the National Institute on Aging (NIA) Genetics of Alzheimer's Disease Data Storage Site (NIAGADS; https://www.nia.nih.gov/research/resource/nia-genetics-alzheimers-disease-data-storage-site-niagads) are not publicly available. Even though many journals might be inclined to accept NIAGADS data as being filed online for scrutiny by the community, detailed authorizations are required before any access can be granted. Most scientists are barred from access. Postdoctoral fellows are not permitted to submit requests for data access: “... investigators must be permanent employees of their institution at a level equivalent to a full-time assistant, associate, or full professor senior scientist... Graduate students and postdoctoral fellows are not permitted to submit project requests” (NIAGADS Website). This runs counter to recommendations (Nightingale and Scott, 2007) that data scrutiny should not be the province of a select elite (“diversity of peer review”), and also to the principles of Open Science.

Identification of a specific person from RNA-seq data is even more difficult than from DNA, and in practice is not achievable without reference to other data. This is in part because our make-up is governed not merely by the genes we have, but by their arrangement on each chromosome—the specific sequence of genetic markers, or “haplotype.” By contrast, all markers in RNA-seq data are “scrambled”—it is not possible to determine which allele the RNA-seq reads derive from. In addition, the tandem repeats held in crime prevention databases (that are not publicly available) are predominantly from untranscribed regions, and are thus not amenable to analysis *via* RNA-seq data. The restrictions on sharing RNA-seq data therefore appear to be unjustified and counter-productive.

## Other types of data

Neuroscience research involves diverse types of anonymized data including brain imaging (e.g., positron emission tomography, PET; magnetic resonance imaging, MRI). However, there is a potential issue with MRI imaging data because the datasets can contain records of facial features, and it is common practice only to share such information once facial imaging has been removed (“de-facing”).

**Figure 1:**
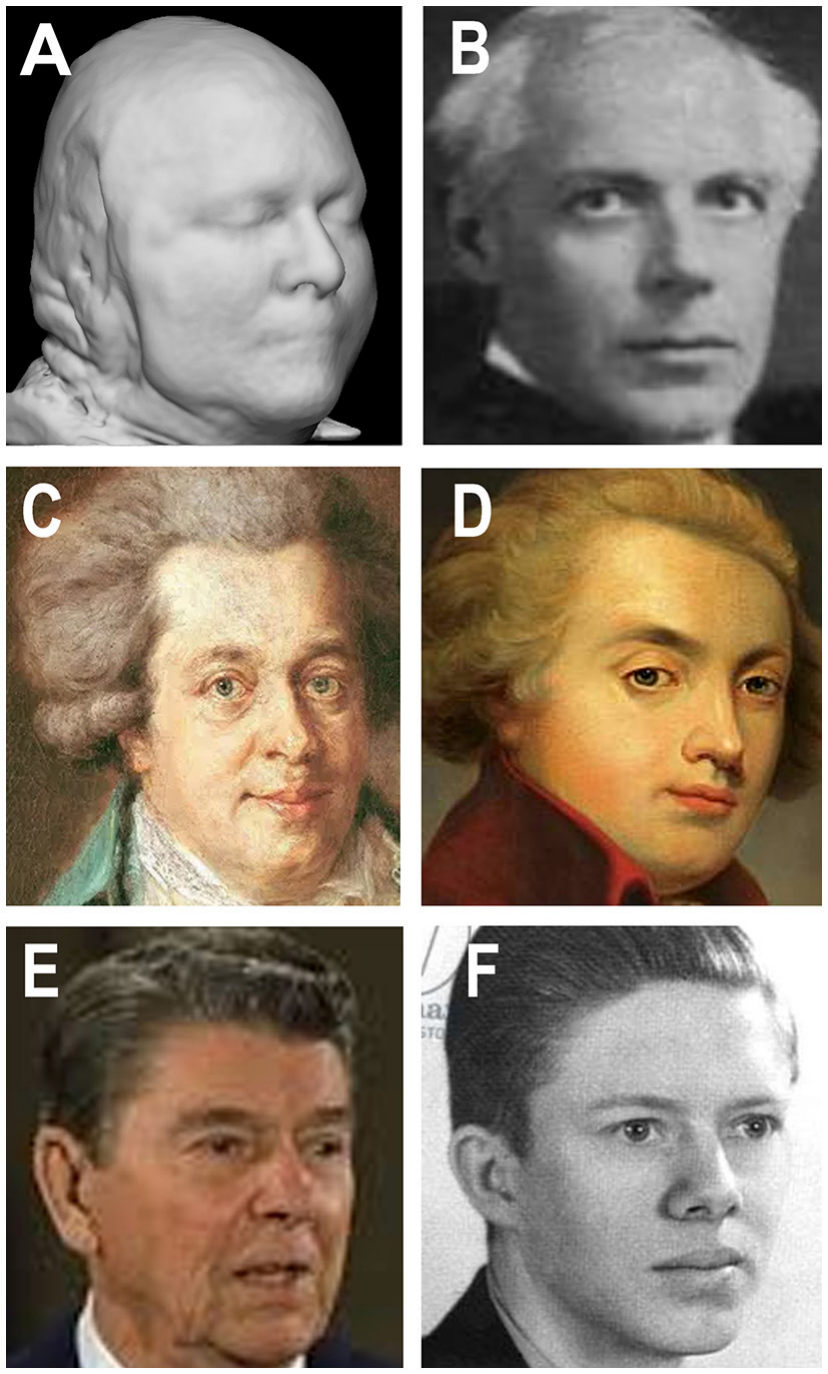
Brain magnetic resonance imaging (MRI) imaging data, composers, presidents, and the vagaries of facial identification. **(A)** Reconstructed image from a modified de-faced MRI image (Schwarz et al., 2021). **(B)** Hungarian composer Béla Bartók (1881–1945) showing similarities to panel **(A)**. **(C, D)** Viennese composer Wolfgang Amadeus Mozart (1756–1791) in two depictions that are poorly similar to each other even though they are of the same person. **(E, F)** American Presidents: Ronald Reagan (1911–2004) and James (Jimmy) Carter (1924–) showing greater or lesser similarity to **(A)**, but millions of other images show equal similarities. The similarities and disparities argue that properly de-faced MRI imaging data, even if partly reconstructed **(A)**, are insufficent to identify any specific individual. **(A)** Reproduced with permission from Christopher Schwarz (Mayo Clinic, Rochester, USA). **(B)** (https://en.wikipedia.org/wiki/B%C3%A9la_Bart%C3%B3k) and **(C)** (https://en.wikipedia.org/wiki/Horn_Concerto_No._1_%28Mozart%29) were granted permission to reproduce under the Wikipedia Creative Commons Attribution-ShareAlike 3.0 Unported License. **(D)** (https://cdn.fansshare.com/photo/wolfgangamadeusmozart/wolfgang-amadeus-mozart-full-image-1140486490.jpg) is reproduced with permission under the FansShare Corporation Copyright and Intellectual Property Policy. **(E)** (https://www.shutterstock.com/editorial/image-editorial/ronald-reagan-396347ci) is reproduced for academic use only with permission from Shutterstock, reference Jeisson 27/12/2022. **(F)** (https://cdn.shopify.com/s/files/1/0720/2785/products/4637.jpeg?v=1571265455) is reproduced under the terms of https://burst.shopify.com/ that grants free reproduction for academic purposes.

Nevertheless, it is possible to reconstruct some details of a participant's facial features using sophisticated computer processing even following de-facing (e.g., Abramian and Eklund, 2019). Schwarz et al. (2021) compared reconstructions to a set of stock images and achieved ~30% correct identifications. The reconstructions provide only a blurry black/white image that is unsatisfactory for unique visual identification (Figure 1), although computer-assisted matching is more accurate. Similar concerns are likely to apply to both MRI and PET imaging (Schwarz et al., 2022). Even so, there is no evidence so far that properly anonymized and de-faced scanning data can be used to identify an individual within a large population (unless the data are already available—C&E).

Other types of physiological data used in the neurosciences include brain recordings such as electroencephalography (EEG) traces, recordings of eye tracking and pupillary metrics, body posture movements, results of psychological tests, among many others. These pose little or no risk—there is no possibility of using such anonymous data to identify an individual.

Looking wider, should details of the microbiome of an individual be “protected”? An international committee states “there are privacy issues because each individual's microbiome is unique” (ALLEA-EASAC-FEAM, 2021).

Given that there is so far no evidence that these potential concerns are empirically based, it is worrying that there has been discussion of potential need to extend the restrictions to other categories of neuroscience data (Eke et al., 2022 for insightful review and further literature).

## The issue of consent

Many individuals voluntarily surrender their rights to privacy by signing a consent form, thereby “donating” their personal data such that investigators can use these data in unraveling the causes of medical conditions and in developing therapies. NIH is seeking a revision to the “Common Rule”—that seeks to protect individuals who participate in research as human subjects (https://nexus.od.nih.gov/all/tag/common-rule/)—by requiring consent to obtained from anyone (providing biological samples) even if the specimens are “deidentified” (Editorial, 2020).

However, this remains a gray area. Even if you and I sign off all our rights, some authorities have ruled that “consent should not be the basis relied upon for processing of personal data...” (discussed by Peloquin et al., 2020). This itself is an enormous issue because database managers will face the issue of determining which entries have secured consent and which have not. As it stands, our formal consent does not necessarily mean that our (anonymous) data will be made available to researchers.

## Should post-mortem data be exempt?

The situation regarding post-mortem data is also unclear. Many datasets online pertain to individuals who passed away years or decades ago. The story of Einstein's brain (https://en.wikipedia.org/wiki/Albert_Einsteins_brain) is of relevance because it could be held that studies thereon (e.g., Men et al., 2014) risk breaching some aspects confidentiality and/or personal data rules, and the case of the HeLa cell line data (Callaway, 2013) should borne in mind.

GDPR recital 27 is that the regulations do not apply to deceased persons (https://gdpr-info.eu/recitals/no-27/), but this is interpreted differently in different countries. In Denmark the Data Protection Act and the GDPR apply to deceased persons until 10 years after the time of death (Article 2, section 5 of the Danish Data Protection Act; https://www.retsinformation.dk/eli/lta/2018/502, in Danish, although after that date some restrictions still apply), whereas in the UK “personal data” means “data relating to a deceased individual where the data would fall within paragraph (a) if it related to a living individual” (https://www.legislation.gov.uk/ukpga/2018/12/schedule/19).

In the USA, the HIPAA protects individually identifiable health information about a decedent for 50 years following the date of death of the individual (https://www.hhs.gov/hipaa/for-professionals/privacy/guidance/health-information-of-deceased-individuals/index.html).

The counter position (following copyright and other rules) is that after a specified number of years it no longer matters whether an individual is or can be identified, and much less so if the data are anonymous. Many of the central issues are discussed in Malgieri (2018). Further consideration should be given to exempting (anonymized) post-mortem data relevant to neuroscience research (perhaps after a suitable delay) from release restrictions. As datasets grow in size year by year, enormous amounts of helpful information are already available for deceased persons, and there is a need to clarify more generally whether these can be released more openly.

## Types of data that could warrant open release

Driven by lack of clarity in the legislation, access to anonymized biomedical data is increasingly restricted. Across the neurosciences and related disciplines, we need to strive harder to make data publicly available, with minimal restrictions on who can access them. There is an urgent, and unmet, need to reassess these data restrictions because “less global sharing of health data for research is hurting everyone” (ALLEA-EASAC-FEAM, 2021), and data governance in the neurosciences “should clarify and simplify the ethical, cultural, and legal issues” (Eke et al., 2022).

For epidemiological data, greater effort will need to be placed on efficient devices such as data minimization and blurring techniques to reduce or eliminate the possibility of individual identification, and speed up release to researchers.

**Figure 2:**
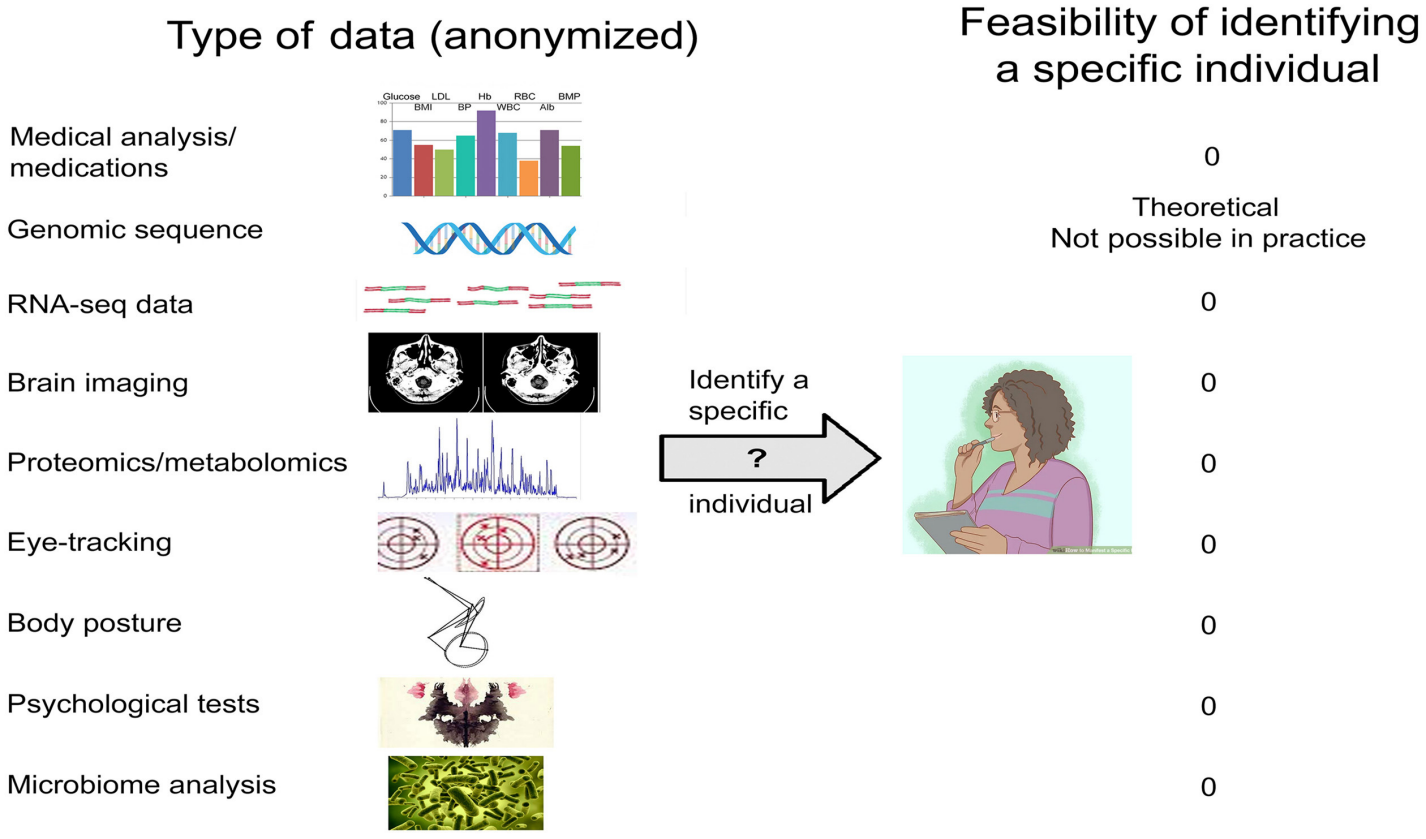
Types of biomedical data relevant to the neurosciences, and the feasibility of identifying a specific individual.

For other types of data (Figure 2), there is no empirical evidence that release of properly anonymized data—be they genomic/transcriptomic, imaging/EEG, eye-tracking, psychological test results, or other—risks identifying specific individuals beyond the C&E and *k*-mer caveats (Table 1). Data that only include age (or age bracket), gender, and disease diagnosis could immediately be used to answer a host of questions such as—(i) are variants in the *APOE* gene a risk factor for neurological disorders; (ii) is virus infection associated with neurodegeneration; (iii) can eye-tracking data be used to diagnose major depression; (iv) is SARS-Co-2 infection associated with motor disturbances; and many other important issues in the field.

**Table 1:** Anonymized biomedical data relevant to the neurosciences that may warrant release without restrictions^a^,^b^.

**Primary data**	**Individual data^c^**		
**Type of data**	**Age^d^**	**Gender**	**Disease diagnosis or control**
Genomic sequence	✓	✓	✓
RNA-seq data	✓	✓	✓
Other “omic” data	✓	✓	✓
Brain imaging (fMRI, CT)^e^	✓	✓	✓
Brain recording (EEG, MEG)	✓	✓	✓
Single-unit recordings	✓	✓	✓
Eye-tracking/pupillometrics	✓	✓	✓
Movement recordings	✓	✓	✓
Psychological tests	✓	✓	✓
Microbiome analysis	✓	✓	✓

Biomedical categories denoted with a tick may warrant release without restrictions.

a The special case of epidemological data (where anonymization may be counterproductive) is discussed in the text.

b Restrictions on post-mortem epidemiological data require further evaluation.

c All other personal data are removed.

d Age in years, or if necessary (and justified), perhaps a 5 year age bracket would be adequate for research purposes.

e De-faced, i.e., removal of facial features.

All types of data are open to use and misuse, but ultimately we need to strike a balance between the benefits of making data openly available to researchers vs. the perceived risk of personal identification. The latter is fraught with uncertainty, and there is a pressing need to evaluate in detail the actual risks of sharing such data among researchers worldwide. In the absence of empirical evidence that the restrictions are valid, the restrictions themselves risk failing the falsifiability test and thus become “unscientific.”

There is also a need for pragmatism. If megacomputers and immense effort are necessary to break the anonymity code, and the risk of harm is minimal, then the benefits are likely to outweigh the risks by a wide margin, and relaxation of global bars on data release would appear to be justified. But, if authoritative assessment determines that the risks are real, and data release could compromise an individual's safety and wellbeing, then we should of course maintain the restrictions. However, if it turns out that our concerns are to a large extent unfounded, then the restrictions should be modified or set aside. Falsifiability and Open Science are not abstruse or optional principles of philosophy (LeBel et al., 2017), they are the bedrock of human rational endeavor.

## Author contributions

The author confirms being the sole contributor of this work and has approved it for publication.
